# Decreased expression of TFF2 and decreased αGlcNAc glycosylation are malignant biomarkers of pyloric gland adenoma of the duodenum

**DOI:** 10.1038/s41598-023-49040-1

**Published:** 2023-12-08

**Authors:** Kazuhiro Yamanoi, Chifumi Fujii, Atsushi Nakayama, Noriko Matsuura, Yusaku Takatori, Motohiko Kato, Naohisa Yahagi, Jun Nakayama

**Affiliations:** 1 Department of Pathology, School of Medicine, Keio University, 35 Shinanomachi, Shinjuku, Tokyo 160-8582 Japan; 2 Department of Molecular Pathology, Shinshu University School of Medicine, Matsumoto, Japan; 3 Department of Biotechnology, Institute for Biomedical Sciences, Interdisciplinary Cluster for Cutting Edge Research, Shinshu University, Matsumoto, Japan; 4 Center for Medical Education and Clinical Training, Shinshu University School of Medicine, Matsumoto, Japan; 5 Division of Research and Development for Minimally Invasive Treatment, Cancer Center, Keio University School of Medicine, Tokyo, Japan

**Keywords:** Surgical oncology, Gastrointestinal diseases

## Abstract

Pyloric gland adenoma (PGA) is a duodenal neoplasm expressing MUC6 and is often associated with high-grade dysplasia and adenocarcinoma. MUC6 secreted from the pyloric gland cells carries unique *O*-glycans exhibiting terminal α1,4-linked *N*-acetylglucosamine residues (αGlcNAc). The small peptide trefoil factor 2 (TFF2) is also secreted from pyloric gland cells and binds to αGlcNAc. We recently demonstrated that αGlcNAc serves as a tumor suppressor for gastric neoplasm including PGA, but the significance of TFF2 expression remains unknown. We examined 20 lesions representing low- and high-grade PGA in 22 cases by immunohistochemistry for αGlcNAc, TFF2, MUC6, MUC5AC, MUC2 and p53. αGlcNAc, TFF2 and MUC6 were co-expressed on the cell surface and a dot-like pattern in the cytosol in low-grade PGA lesions. High-grade PGA also expressed MUC6, but reduced αGlcNAc and TFF2 expression. The ratios of αGlcNAc or TFF2 to MUC6 score in high-grade PGA were significantly lower than low-grade PGA (*P* < 0.001). Co-expression of αGlcNAc-glycosylated MUC6 and TFF2 in PGA suggests the existence of αGlcNAc/TFF2 form complex in PGA cells, a finding consistent with our observations in non-neoplastic Brunner’s gland cells. The decreased αGlcNAc and TFF2 expression are associated with high grade atypical cells, indicative of the malignant potential of PGA.

## Introduction

Detection of duodenal neoplasms has improved due to use of gastrointestinal endoscopy^1^. Duodenal neoplasms show intestinal or gastric gland differentiation, and among the latter, pyloric gland adenoma (PGA) is the major subtype and is highly positive for MUC6 and more variably for MUC5AC^2–4^. PGAs are also seen in stomach. In both stomach and duodenum, PGA exhibits a notable frequency of *GNAS* or *KRAS* mutations, suggesting linkage of these mutations and distinguishing PGA from other types of adenomas^5^. PGAs are classified as low-grade and high-grade subtypes, based on cellular atypia^3,4^. PGA often exhibits high-grade dysplasia and is reportedly associated with a high risk of malignant transformation to adenocarcinoma^3,4^. Previous studies reported that 30% of PGA cases showed transition to well differentiated adenocarcinoma at the time of diagnosis^2^, and 12% are associated with intramucosal or rather invasive adenocarcinomas^4^. Accordingly, it is critical for pathologists to diagnose PGA correctly before development of adenocarcinoma. However, identifying histological atypia in PGA is sometimes difficult, making a correct diagnosis is difficult.

*O*-glycans attached to the mucin core peptide MUC6 and containing terminal α1,4-linked *N*-acetylglucosamine residues, hereafter designated αGlcNAc, are unique to gland mucin secreted from mucous neck cells and pyloric gland cells of the gastric mucosa and form Brunner’s gland of the duodenal mucosa^6^. Previously, we used expression cloning to isolate cDNA encoding α1,4-*N*-acetylglucosaminyltransferase (α4GnT), the enzyme catalyzing αGlcNAc biosynthesis^7^, and showed that αGlcNAc functions as a natural antibiotic to protect gastric mucosa from *Helicobacter pylori (H. pylori)* infection^8^.

We also previously generated knockout mice deficient in *A4gnt*, which encodes α4GnT, to assess αGlcNAc function *in vivo*^9^. Mutant mice completely lacked αGlcNAc expression in the gastroduodenal mucosa, indicating that α4GnT is the sole enzyme responsible for αGlcNAc biosynthesis^9^. Significantly, mutant mice developed gastric adenocarcinoma through a hyperplasia-dysplasia-carcinoma sequence, even in the absence of *H. pylori* infection, indicating that αGlcNAc is directly associated with gastric tumorigenesis, independent of *H. pylori* infection^10^.

In addition, we previously showed that reduced αGlcNAc glycosylation relative to MUC6 levels was associated with malignant potential of both adenoma and carcinoma with pyloric mucin phenotypes in stomach, pancreas, bile duct and uterine cervix^11–16^. Recently, we ectopically expressed α4GnT*,* the αGlcNAc biosynthetic enzyme, together with MUC6 in two human pancreatic cancer cell lines and observed significantly suppressed proliferation in both lines relative to controls^17^. Moreover, cellular motility decreased following MUC6 ectopic expression, an effect enhanced by co-transduction with α4GnT. MUC6 expression also attenuated invasiveness of both lines relative to controls, and this effect was also enhanced by additional α4GnT expression^17^. Furthermore, we found αGlcNAc-glycosylated MUC6 formed a complex with trefoil factor 2 (TFF2) *in vitro*^17^.

Trefoil factors (TFFs) are a group of stable polypeptides with a molecular weight of 6–12 kDa, which are secreted from mucin-secreting cells of the mammalian gastrointestinal epithelium including human and mice^18–21^. TFF2 is also known as spasmolytic polypeptide expressed in intestinal metaplasia of human stomach^22,23^.

H^+^/K^+^-ATPase (a major product of parietal cells), pepsinogen-1 (a major secretory product of chief cells and mucous neck cells) and MIST1 (a transcription factor specific to chief cells) are well known as gastric gland markers. Also, they are expressed in gastric adenocarcinoma of the fundic gland type^25,26^.

In the present study, we examined expression patterns of TFF2, αGlcNAc, p53 and mucin core proteins MUC6 MUC5AC and MUC2 in PGA of human duodenum by immunohistochemistry and then compared relative expression of TFF2, αGlcNAc and MUC6 in low- and high-grade PGAs. We observed expression of both αGlcNAc and TFF2 in low-grade PGA and decreased expression of both in high-grade PGA, the latter associated with malignant transformation of PGA. Furthermore, we examined expression patterns of gastric fundic gland markers including H^+^/K^+^-ATPase, pepsinogen-1 and MIST1.

## Materials and methods

### Patients

The present study evaluated tissue samples from 22 patients with non-ampullary duodenal mucosal neoplasia with pyloric gland differentiation in Keio University Hospital. All specimens were obtained by endoscopic submucosal dissection (ESD) and fixed in 10% buffered formalin and then paraffin-embedded. Pathological diagnosis of pyloric gland adenoma (PGA) was performed based on Hematoxylin and Eosin (H&E)-stained sections, according to WHO classification criteria^23^. PGA was categorized as low-grade or high-grade; briefly, presence of a high nucleus/cytoplasm ratio, prominent nucleoli, numerous mitoses, nuclei extending into the lumen, and/or loss of nuclear polarity of the tumor cells was indicative of high-grade^24^. Most PGA specimens contain a continuum of high-grade and low-grade lesions. By reviewing H&E-stained sections, the most obvious low-grade and/or high-grade lesions in each case were selected for further analysis. Clinical characteristics of 22 PGA cases are shown in Table 1.

**Table 1:** Clinical characteristic of 22 duodenal pyloric adenoma cases.

Mean age (range)	69.7 (56–85)
Male/female	7/15
Mean diameter (range)(mm)	21.6 (8–55)
Lesion number (high/low grade)	20/20

### Immunohistochemistry

Immunohistochemical staining was performed using a Bond-MAX Automated Stainer (Leica Biosystems, Wetzlar, Germany). Before immunostaining, antigen retrieval was carried out in 10 mM Tris–HCl buffer (pH 9.0) containing 1 mM EDTA. The primary antibodies used for immunohistochemistry were anti-αGlcNAc (clone HIK1083, mouse IgM, Kantokagaku, Tokyo, Japan) diluted 1/100, anti-MUC2 (clone Ccp58, mouse IgG, Novocastra, Newcastle, UK) diluted 1/200, anti-MUC5AC (clone CLH2, mouse IgG, Novocastra) diluted 1/100, anti-MUC6 (clone CLH5, mouse IgG, Novocastra) diluted 1/200, anti-TFF2 (clone 2A10, Novus Biologicals, Centennial, CO, US) diluted 1/200, anti-p53 (clone DO-7, mouse IgG, DAKO) diluted 1/2000, anti-MIST-1 (clone D7N4B, rabbit IgG; Cell Signaling Technology, Danvers, MA, USA) diluted 1/100, anti-pepsinogen-1 (clone 8003(99/12), mouse IgG; Bio-Rad, Hercules, CA, USA) diluted 1/100, and anti-H^+^/K^+^-ATPase (clone 1H9, mouse IgG; MBL, Nagoya, Japan) diluted 1/100. Negative control experiments were done by omitting primary antibodies from the immunostaining procedure, and no specific staining was found.

Double immunohistochemistry using anti-TFF2 and anti-αGlcNAc was performed using a Bond-MAX Automated Stainer. TFF2 was detected by using DAB, a horseradish peroxidase-based system (ImmPress; Vector Laboratories, Burlingame, CA, USA) and αGlcNAc was detected using Fast Red, an alkaline phosphatase-based system (N-Histofine Simple Stain, Nichirei Bioscience, Tokyo, Japan).

### Semi-quantification of mucin core protein, αGlcNAc, TFF2, MIST-1, pepsonigen-1 and H^+^/K^+^-ATPase expression

Evaluation of expression of mucin core proteins and of αGlcNAc and TFF2 was carried out based on the proportion of positively-stained neoplastic cells to the total number of neoplastic cells in each low- or high-grade PGA lesions, and then graded from 0 to 3, where 0 represents < 10% of tumor cells; 1 represents 11–33% of tumor cells; 2 represents 33–66% of tumor cells; and 3 represents greater than 66% of tumor cells, as described previously^14–16^. The ratio of αGlcNAc or TFF2 to MUC6 for all 40 lesions was then calculated.

The results of p53 staining were classified into diffuse, wild-type, and loss of expression. The wild-type pattern is characterized by weak heterogeneous expression.

MIST-1, pepsinogen-1 and H^+^/K^+^-ATPase expression were evaluated on the proportion of positively-stained neoplastic cells to the total number of neoplastic cells in all PGA lesions including low and high, and then graded from 0 to 3, described previously^14–16^.

### Statistics

Semi-quantitative expression scores for each mucin marker MUC5AC, MUC6 and αGlcNAc and for TFF2 were analyzed statistically using the Wilcoxon matched parts test. Difference in the ratios of TFF2 or αGlcNAc to MUC6 expression score between high-grade and low-grade PGA lesions was also analyzed. *P* values < 0.05 were considered statistically significant. All analyses were carried out using Microsoft Office Excel 2007 software (Microsoft Corporation, Redmond, WA, USA). Violin plots were created by using JMP software (JMP Statistical Discovery LLC, Cary, NC, USA).

### Ethical approval

The study was performed in accordance with the Declaration of Helsinki and approved by the Ethical Committees of the Keio University School of Medicine, Tokyo, Japan (no. 20180074). Written informed consent was obtained from all patients.

## Results

### Expression of mucin markers and p53

Based on criteria outlined in Methods, we selected low grade PGA lesions (20 lesions) and high-grade PGA lesions (20 lesions) in 22 duodenal ESD specimens (Supplementary Table 1).

**Figure 1:**
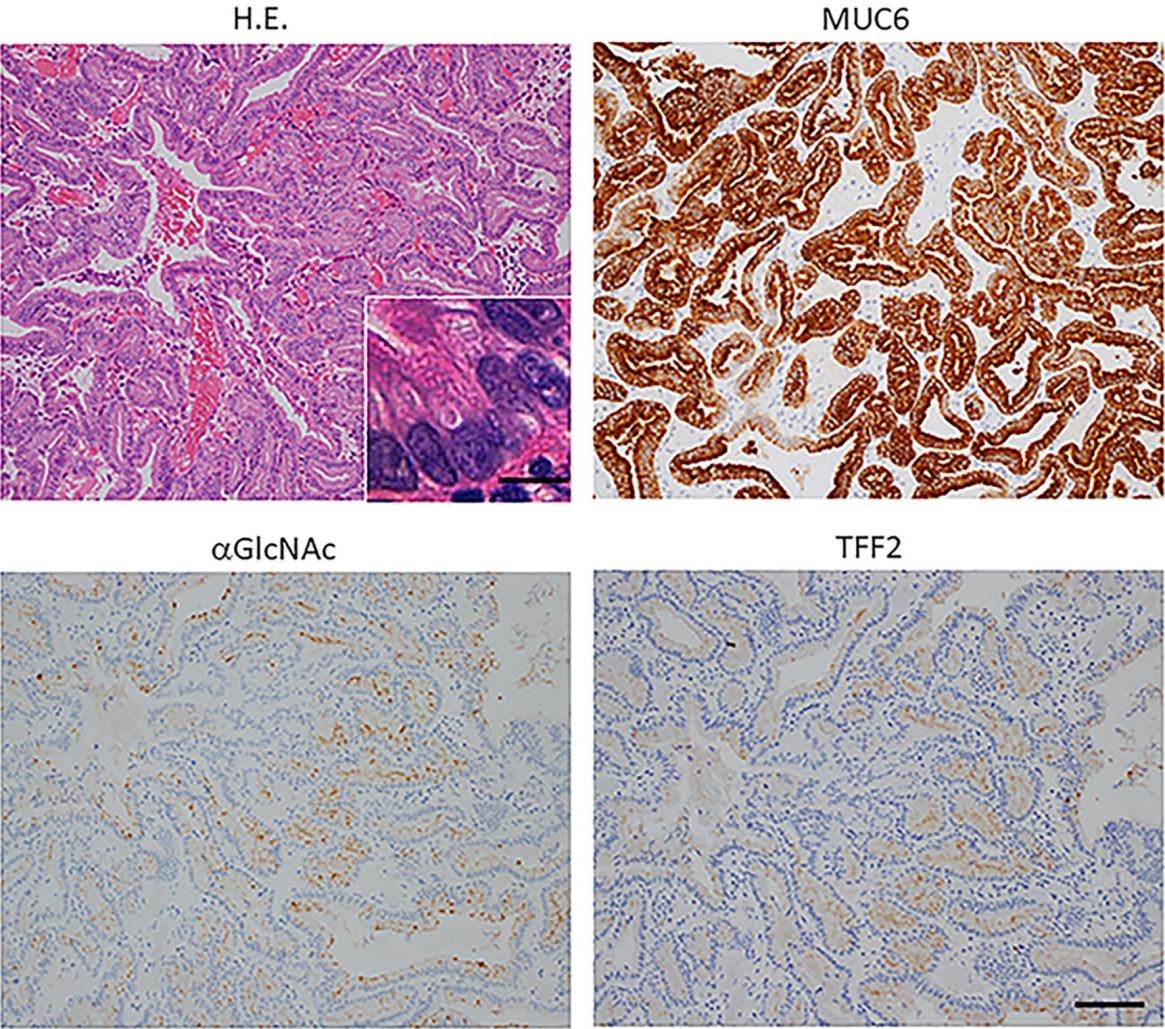
Immunohistochemical detection of MUC6-, αGlcNAc-, and TFF2-positive cells in low grade PGA. The inset shows an enlarged view of a H&E section. H&E staining shows cell polarity is preserved and nucleolar stratification is not evident, indicating low-grade PGA lesion (case No.20 in Supplementary Table 1). Immunohistochemistry of serial tissue specimen with antibodies shows that tumor cells are positive for antibodies against MUC6, αGlcNAc and TFF2. Scale bar: 100 µm. Inset scale bar: 10 µm.

**Figure 2:**
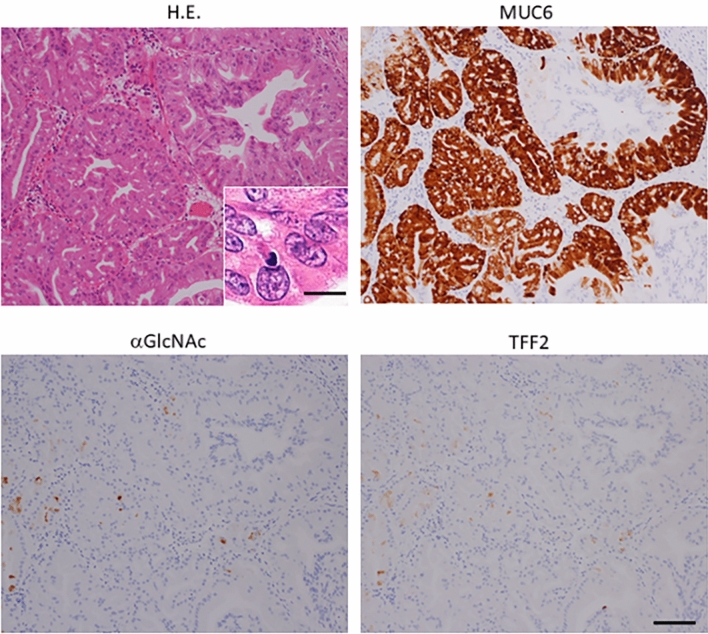
Immunohistochemical detection of MUC6-, αGlcNAc-, and TFF2-positive cells in high-grade PGA. The inset shows an enlarged view of a H&E section. H&E staining shows loss of cell polarity and evident nucleolus, indicating high grade PGA lesion (case No.4 in Supplementary Table 1). Immunohistochemistry against MUC6 antibodies indicates broad positivity. By contrast, αGlcNAc, and TFF2 staining is reduced relative to that of MUC6. Scale bar: 100 µm. Inset scale bar: 10 µm.

**Figure 3:**
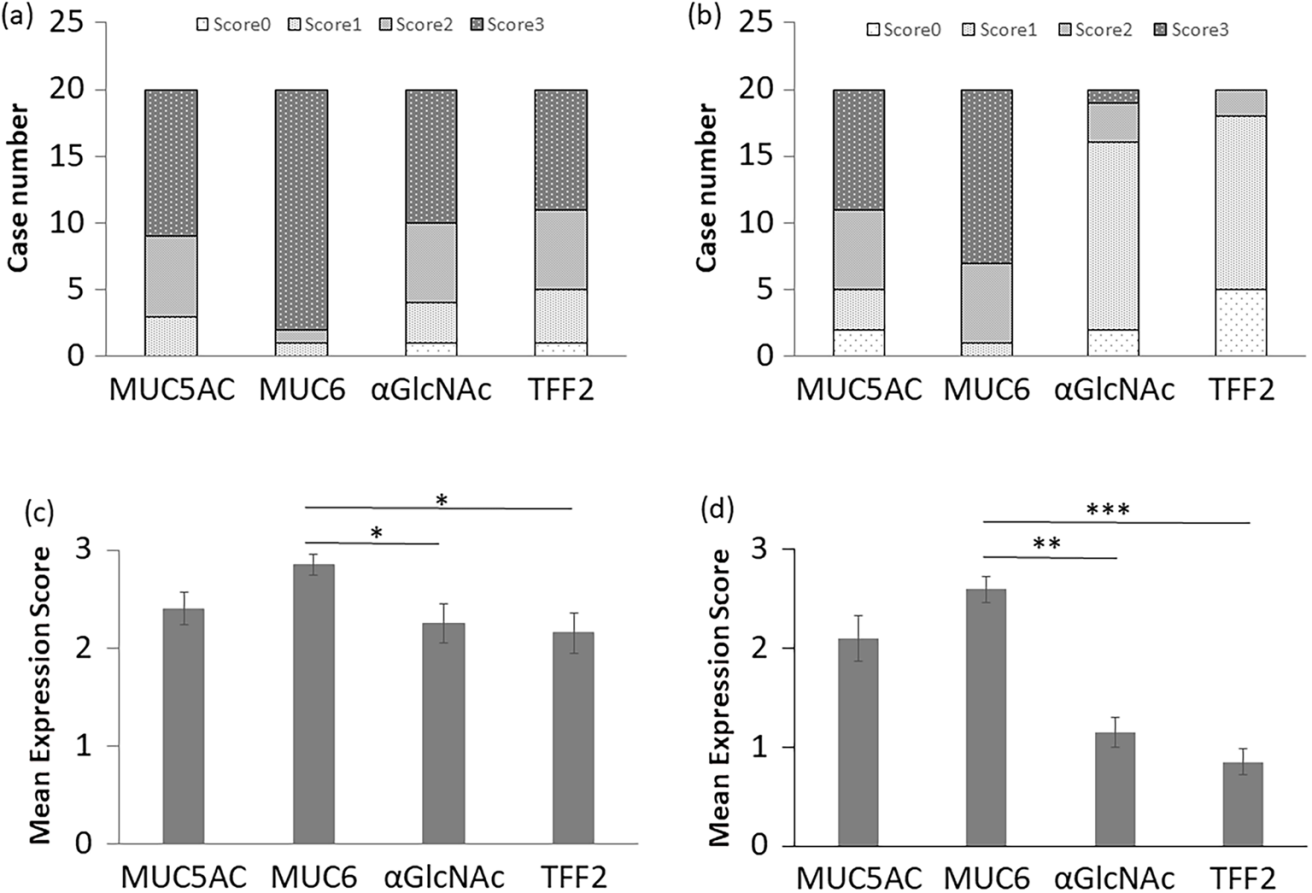
Semi-quantitative analysis of MUC5AC, MUC6, αGlcNAc and TFF2 expression scores in low- and high-grade PGA. Bar graph indicates case numbers of each score in low- (**a**) or high-grade (**b**) PGA. Differences in MUC5AC, MUC6 and αGlcNAc expression scores in low-(**c**) or high-grade (**d**) PGA are compared. Data are represented as means ± SEM (**c**, **d**).

**Figure 4:**
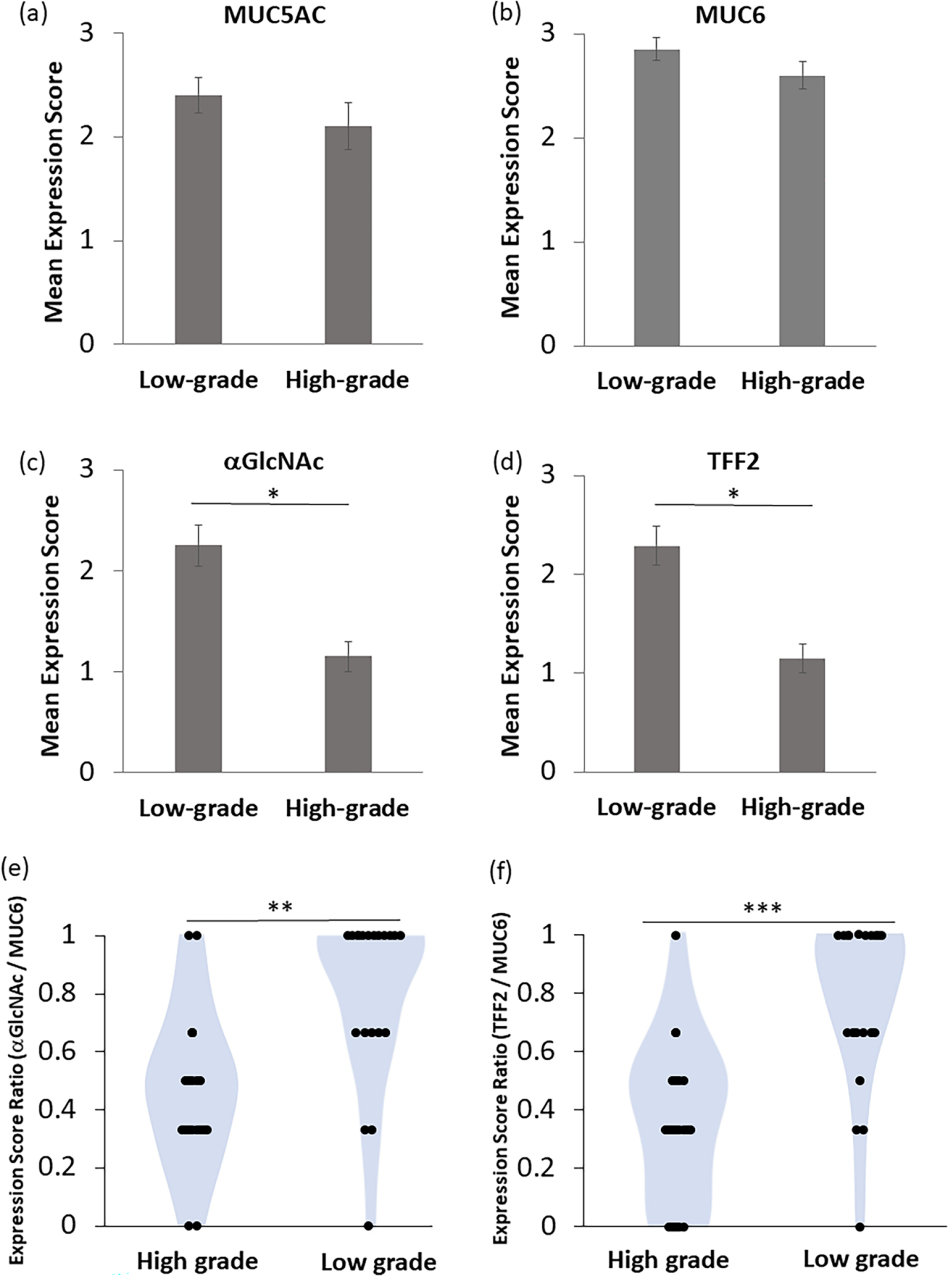
Differences in MUC5AC (**a**), MUC6 (**b**), αGlcNAc (**c**), TFF2 (**d**), the ratios of αGlcNAc to MUC6 (**e**) or the ratios of TFF2 to MUC6 (**f**) expression scores between low- and high-grade PGA are compared. Data are represented as means ± SEM (**a**–**d**) and as violin plot (**e**, **f**). *P < 0.01, **P < 0.001 ***P < 0.0001 by the Wilcoxon matched-pair test (**a**–**f**).

Immunohistochemical analysis of both low- and high-grade lesions showed MUC6 expression in tumor cells irrespective of histological grade (Figs. 1, 2, 3a,b). Also, all low-grade PGA lesions were MUC5AC-positive, while 18 of 20 high-grade lesions were MUC5AC-positive (Fig. 3a,b). We then compared MUC6 and MUC5AC expression scores in low- and high-grade PGA. In low-grade, the MUC6 expression score was 3 in 18 cases, 2 in 1 case, and 1 in 1 case (Fig. 3a and Supplementary Table 1), while in high-grade, the MUC6 score was 3 in 13 cases, 2 in 6 cases, and 1 in 1 case (Fig. 3b and Supplementary Table 1). In low-grade PGA, the MUC5AC expression score was 3 in 11 cases, 2 in 6 cases, 1 in 3 cases (Fig. 3a and Supplementary Table 1), while in high-grade, it was 3 in 9 cases, 2 in 6 cases, 1 in 3 cases, and 0 in 2 cases (Fig. 3b and Supplementary Table 1). We then performed a statistical comparison between MUC5AC and MUC6 scores in both low-grade and high-grade PGA lesions but did not observe a significant difference for either lesion (*P* = 0.0729 for low-grade PGA lesions, *P* = 0.0917 for high-grade PGA lesions) (Fig. 3c,d). Moreover, when we compared differences in MUC5AC or MUC6 scores between low- and high-grade PGA lesions, there were no significant differences in MUC5AC or MUC6 expression scores (*P* = 0.292 for MUC5AC, *P* = 0.187 for MUC6) (Fig. 4a,b).

MUC2 is a mucin protein specifically expressed in normal intestine and in tumors with intestinal phenotypes^2,3^. In contrast to findings relevant to MUC5AC and MUC6, all low- and high-grade PGA lesions were MUC2-negative (Supplementary Table 1). Moreover, in all low- and high-grade PGA lesions, p53 expression was classified as wild-type (Supplementary Table 1). In summary, these expression patterns of mucin core proteins MUC5AC, MUC6 and MUC2 and of p53 were generally consistent with those previously reported^2,3^.

### Expression of αGlcNAc and TFF2

In low-grade lesions, αGlcNAc and TFF2 were positively expressed in the same 19 of 20 lesions in low-grade PGA. αGlcNAc and TFF2 scored 3 in 10 cases, αGlcNAc scored 2 in 6 cases, TFF2 scored 2 in 5 cases, αGlcNAc scored 1 in 3 cases and TFF2 scored 1 in 4 cases (Fig. 3a and Supplementary Table 1). In high-grade lesions, 18/20 were αGlcNAc positive and 15/20 were TFF2 positive (Fig. 3b and Supplementary Table 2). In all lesions positive for both αGlcNAc and TFF2, areas showing αGlcNAc expression generally corresponded to those expressing TFF2 (Figs. 1, 2). We observed no differences in αGlcNAc and TFF2 expression scores in low- or high-grade PGA (*P* = 0.317 for low-grade PGA; *P* = 0.0679 for high-grade PGA) (Figs. 3c,d)

We then assessed expression scores between MUC6 and αGlcNAc and between MUC6 and TFF2. In both low- and high-grade PGA lesions, the MUC6 score was significantly higher than that of αGlcNAc. (*P* = 0.00965 for low-grade; *P* = 1.35x10^-4^ for high-grade) (Fig. 3c,d). Also in both low- and high-grade PGA lesions, the MUC6 expression score was higher than that of TFF2 (*P* = 0.00506 for low-grade; *P* = 9.44x10^-5^ for high-grade) (Fig. 3c,d). Furthermore, the difference in mean expression score for αGlcNAc and MUC6 in high-grade PGA was greater than that seen in low-grade PGA. Moreover, differences between mean expression scores of TFF2 and MUC6 in high-grade PGA were greater than that those seen in low-grade (Fig. 3c,d). When we compared differences in αGlcNAc or TFF2 expression scores in low- and high-grade PGA, individual αGlcNAc and TFF2 expression scores in low-grade PGA were significantly higher than those seen in high-grade. (*P* = 0.00532 for αGlcNAc; *P* = 0.00192 for TFF2) (Fig. 4c,d). Finally, we calculated the ratio of αGlcNAc or TFF2 to MUC6 expression scores in all lesions (Supplementary Tables 3 and 4). We then compared the differences of these ratios between in low-grade and high-grade PGA lesions. The ratios of αGlcNAc or TFF2 to MUC6 in low-grade PGA were significantly higher than those seen in high-grade PGA (*P* = 3.90x10^-4^ for the ratio of αGlcNAc to MUC6; *P* = 4.29 × 10^−5^ for the ratio of TFF2 to MUC6) (Fig. 4e,f).

### αGlcNAc and TFF2 expression is comparable in low grade PGA

**Figure 5:**
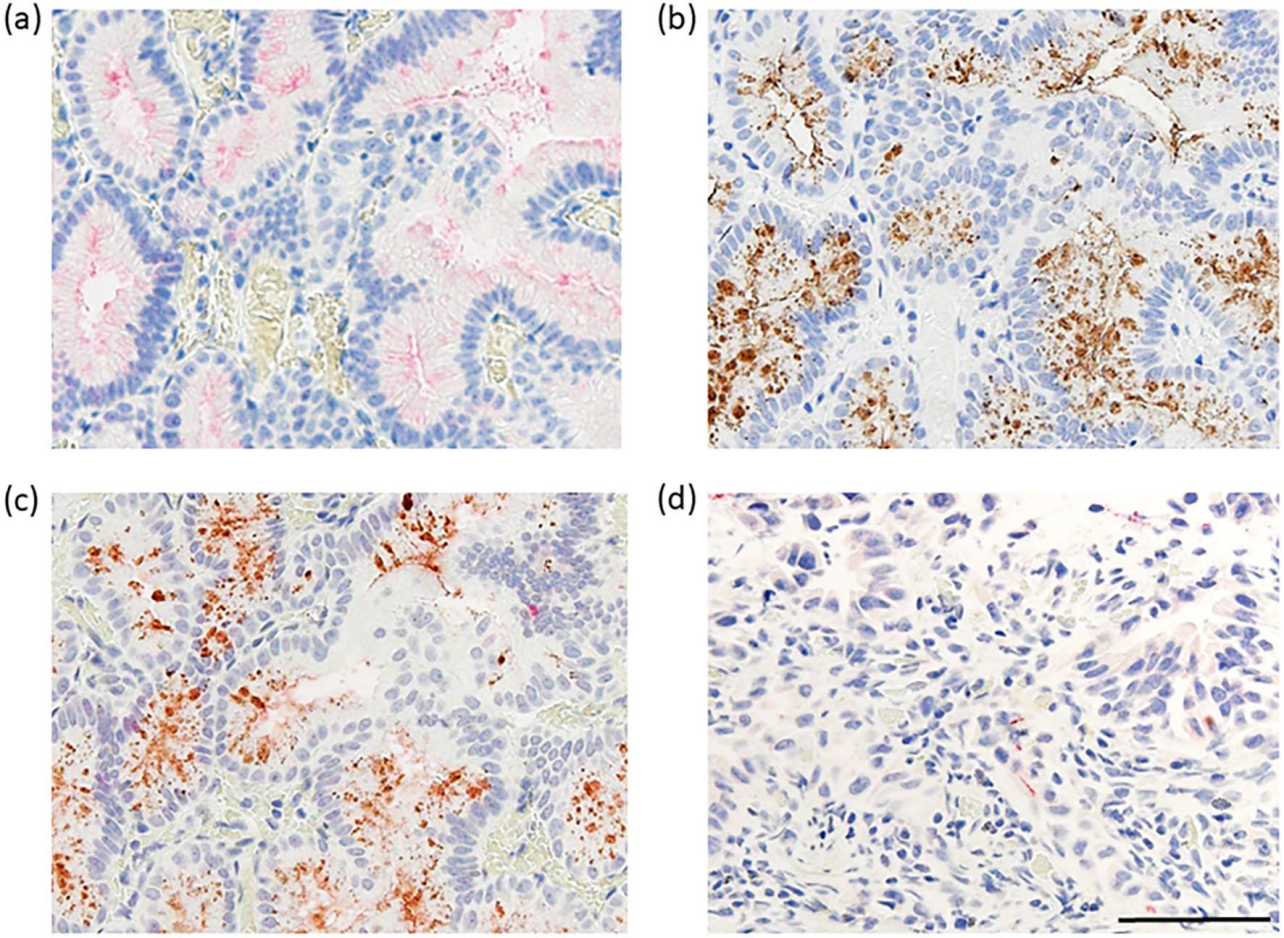
Immunohistochemical expression of αGlcNAc, and TFF2 in a low-grade PGA lesion (case 19) (**a**–**c**) and a high-grade PGA lesion (case 13) (**d**). Single immunohistochemical analysis of αGlcNAc using Fast Red staining (**a**). Single immunohistochemical analysis of TFF2 based on DAB staining (**b**). Double immunohistochemistry of αGlcNAc and TFF2 (**c**, **d**). Both GlcNAc and TFF2 are expressed in low-grade PGA lesion (**c**). In high-grade PGA lesion, both expressions are lost (**d**). Scale Bar: 100 μm.

Generally, expression levels and cellular location of αGlcNAc and TFF2 are comparable in most cells analyzed (Fig. 1). Thus we used dual immunohistochemical staining of a case harboring a low-grade PGA lesion to ask whether αGlcNAc and TFF2 exhibited similar expression patterns. Analysis of that lesion revealed red-stained material indicative of αGlcNAc positivity and brown-colored material indicative of TFF2 positivity at the apical cell surface and in a dot-like pattern in the cytosol (Fig. 5a,b). Staining generally co-localized in dual-colored sections (Fig. 5c). In a high-grade PGA lesion, both αGlcNAc and TFF2 expressions were lost in dual immunohistochemically staining (Fig. 5d).

### Gastric fundic gland markers are focally expressed in PGA

Pepsinogen-1 was positively expressed in 6 of 22 PGAs. Score 1 in 4 PGAs, score 2 in 1 PGA and score 3 in 1 PGAs. H^+^/K^+^-ATPase was positively expressed in 4 of 22 PGAs. Score 1 in 3 PGAs, score 2 in 1 PGA. MIST-1 was positively expressed in 14 of 22 PGA cases. Score 1 in 6 PGAs, score 2 in 6 PGAs and score 3 in 2 PGAs (Supplementary Table 5 and Fig. 2).

## Discussion

In the present study, we first confirmed gastric mucin-specific MUC6 and MUC5AC expression, but not that of intestinal mucin-specific MUC2, in all PGA tumors assessed, and showed that αGlcNAc and TFF2 were diffusely co-expressed in low-grade PGA, patterns also seen in normal Brunner’s glands (Fig. 1). However, both αGlcNAc and TFF2 expressions are decreased in high-grade dysplasia of PGA (Fig. 2). The ratios of αGlcNAc or TFF2 to MUC6 expression levels were significantly decreased in high-grade PGA. Finally, differences between MUC6 and either TFF2 or αGlcNAc expression scores in high-grade PGAs were greater than those seen in low-grade ones (Figs. 3, 4).

We previously reported that decreased αGlcNAc glycosylation of MUC6 was associated with high mitotic activity in tumor cells, indicative of the malignant potential of gastric PGA^12^. Kushima et al. also reported that PGAs invariably exhibited diffuse MUC6 and TFF2 expression^25^. However, they did not discuss the difference in expression of these markers between low-grade and high-grade PGAs. Our present data and that reported previously indicate that reduced expression of either TFF2 or αGlcNAc relative to MUC6 could serve as a biomarker of malignant potential in PGA of stomach and duodenum.

We previously observed decreased αGlcNAc glycosylation of MUC6 in cancers of the pancreas, lung, common bile duct and uterine cervix^13–16^. Furthermore, our recent in vitro analysis of pancreatic cancer lines showed that MUC6 expression significantly suppressed cancer cell proliferation, motility and invasiveness, and that αGlcNAc-glycosylated MUC6 had significantly more robust anti-cancer effects^17^.

TFF2 binds MUC6 through αGlcNAc to form a complex that increases viscosity of mucus in the gastric mucosa^27–29^. Thus, we previously focused on TFF2 expression in cells expressing αGlcNAc-glycosylated MUC6^17^. In that study, we detected glycosylated MUC6 in culture supernatants and confirmed the presence of a complex of αGlcNAc-glycosylated MUC6 and TFF2 in those supernatants by immunoprecipitation^17^.

In the present study we used dual immunohistochemistry to show that TFF2/αGlcNAc co-localize not only on the cell surface but in a dot-like pattern in the cytosol in low-grade PGA cells from human duodenal specimens (Fig. 5). We also observed diffuse co-expression of TFF2 and αGlcNAc in non-neoplastic Brunner’s gland cells (Supplementary Fig. 1). Hoffman previously showed that condensed and highly organized forms of MUC6 are found in a complex with TFF2 in secretory granules^30^. Our results support the idea that αGlcNAc-glycosylated MUC6 rapidly forms a complex with TFF2 in secretory granules and once those granules move to the cell surface, these factors remain in a complex. In high-grade PGAs, both TFF2 expression and αGlcNAc glycosylation decreased relative to low-grade PGAs (Figs. 1, 2). These data are consisted with that TFF2 are found in a complex with αGlcNAc-glycosylated MUC6 indicated as reported previously^17,30^.

PGA is a major gastric phenotype neoplasm arising in the duodenum, and reportedly has the malignant potential to develop into adenocarcinoma^1–3^. These studies indicate that PGA is derived from mucous cells in pyloric or gastric mucous glands that express αGlcNAc-glycosylated MUC6 in a complex with TFF2. Seven of 22 PGA cases exhibited focal expression of pepsinogen-1, 4 had focal H^+^/K^+^-ATPase and 15 had focal MIST1 expression, suggesting that PGAs have also focal gastric oxyntic glands phenotype. Kushima et al. reported that 10 of 12 PGAs also unexpectedly exhibited focal expression of pepsinogen-1 and MIST1, suggesting that PGAs often show focal chief cell differentiation and phenotypically resemble mucous neck cells rather than pyloric glands^25^. Our present data also supports their suggestion that PGAs resembles gastric mucous neck cell phenotype.

In conclusion, decreased αGlcNAc glycosylation on the MUC6 scaffold protein also decreases TFF2 levels in that complex and could serve as an indicator of relatively high-grade PGA progressing to adenocarcinoma. Thus, immunohistochemistry for TFF2 and αGlcNAc could be useful to diagnose PGA in routine pathology assessment of gastroduodenal specimens.

### Supplementary Information

Supplementary Information 1.

Supplementary Information 2.

## Data Availability

Data is available from the corresponding author upon reasonable request.

## References

[1] Goda K, Kikuchi D, Yamamoto Y (2014). Endoscopic diagnosis of superficial non-ampullary duodenal epithelial tumors in Japan: Multicenter case series. Dig. Endosc..

[2] Vieth M, Kushima R, Borchard F, Stolte M (2003). Pyloric gland adenoma: A clinico-pathological analysis of 90 cases. Virchows Arch..

[3] Kushima R, Vieth M, Borchard F (2006). Gastric-type well-differentiated adenocarcinoma and pyloric gland adenoma of the stomach. Gastric Cancer.

[4] Chen ZM, Scudiere JR, Abraham SC, Montgomery E (2009). Pyloric gland adenoma: An entity distinct from gastric foveolar type adenoma. Am. J. Surg. Pathol..

[5] Matsubara A, Sekine S, Kushima R (2013). Frequent GNAS and KRAS mutations in pyloric gland adenoma of the stomach and duodenum. J. Pathol..

[6] Zhang MX, Nakayama J, Hidaka E (2001). Immunohistochemical demonstration of α1,4-*N*-acetylglucosaminyltransferase that forms GlcNAcα1,4Galβ residues in human gastrointestinal mucosa. J. Histochem. Cytochem..

[7] Nakayama J, Yeh J-C, Misra AK (1999). Expression cloning of a human α1,4-*N*-acetylglucosaminyltransferase that forms GlcNAcα1→4Galβ→R, a glycan specifically expressed in the gastric gland mucous cell-type mucin. Proc. Natl. Acad. Sci. USA.

[8] Kawakubo M, Ito Y, Okimura Y (2004). Natural antibiotic function of a human gastric mucin against *Helicobacter pylori* infection. Science.

[9] Karasawa F, Shiota A, Goso Y (2012). Essential role of gastric gland mucin in preventing gastric cancer in mice. J. Clin. Invest..

[10] Nakayama J (2014). Dual roles of gastric gland mucin-specific *O*-glycans in prevention of gastric cancer. Acta Histochem. Cytochem..

[11] Shiratsu K, Higuchi K, Nakayama J (2014). Loss of gastric gland mucin-specific O-glycan isassociated with progression of differentiated-type adenocarcinoma of the stomach. Cancer Sci..

[12] Yamanoi K, Sekine S, Higuchi K, Kushima R, Nakayama J (2015). Decreased expression of gastric gland mucin-specific glycan α1,4-linked *N*-acetylglucosamine on its scaffold mucin 6 is associated with malignant potential of pyloric gland adenoma of the stomach. Histopathology.

[13] Yamada S, Yamanoi K, Sato Y, Nakayama J (2020). Diffuse MIST1 expression and decreased αGlcNAc glycosylation on MUC6 are distinct hallmark for gastric neoplasms exhibiting oxyntic gland differentiation. Histopathology.

[14] Ohya A, Yamanoi K, Shimojo H, Fujii C, Nakayama J (2017). Gastric gland mucin-specific *O*-glycan expression decreases with tumor progression from precursor lesions to pancreatic cancer. Cancer Sci..

[15] Yamanoi K, Ishii K, Tsukamoto M, Asaka S, Nakayama J (2018). Gastric gland mucin-specific O-glycan expression decreases as tumor cells progress from lobular endocervical gland hyperplasia to cervical mucinous carcinoma, gastric type. Virchows Arch..

[16] Okumura M, Yamanoi K, Uehara T, Nakayama J (2020). Decreased alpha-1,4-linked *N*-acetylglucosamine glycosylation in biliary tract cancer progression from biliary intraepithelial neoplasia to invasive adenocarcinoma. Cancer Sci..

[17] Yuki A, Fujii C, Yamanoi K (2022). Glycosylation of MUC6 by α1,4-linked *N*-acetylglucosamine enhances suppression of pancreatic cancer malignancy. Cancer Sci..

[18] Cook GA, Familari M, Thim L, Giraud AS (1999). The trefoil peptides TFF2 and TFF3 are expressed in rat lymphoid tissues and participate in the immune response. FEBS Lett..

[19] Mihalj M, Bujak M, Butković J (2019). Differential expression of TFF1 and TFF3 in patients suffering from chronic rhinosinusitis with nasal polyposis. Int. J. Mol. Sci..

[20] Viby NE, Nexø E, Kissow H (2015). Trefoil factors (TFFs) are increased in bronchioalveolar lavage fluid from patients with chronic obstructive lung disease (COPD). Peptides.

[21] Popp J, Schicht M, Garreis F (2019). Human synovia contains trefoil factor family (TFF) peptides 1–3 although synovial membrane only produces TFF3: Implications in osteoarthritis and rheumatoid arthritis. Int. J. Mol. Sci..

[22] Nam KT, Lee HJ, Sousa JF (2010). Mature chief cells are cryptic progenitors for metaplasia in the stomach. Gastroenterology.

[23] Weis VG, Sousa JF, LaFleur BJ (2013). Heterogeneity in mouse spasmolytic polypeptide- expressing metaplasia lineages identifies markers of metaplastic progression. Gut.

[24] Lauwers GY, Carnerio F, Graham DY, Bosman FT, Carneiro F, Hruban RH, Theise ND (2010). Gastric carcinoma. WHO Classification of Tumours of the Digestive System.

[25] Kushima R, Sekine S, Matsubara A (2013). Gastric adenocarcinoma of the fundic gland type shares common genetic and phenotypic features with pyloric gland adenoma. Pathol. Int..

[26] Ueyama H, Yao T, Nakashima Y (2010). Gastric adenocarcinoma of fundic gland type (chief cell predominant type): Proposal for a new entity of gastric adenocarcinoma. Am. J. Surg. Pathol..

[27] Kjellev S, Nexø E, Thim L, Poulsen SS (2006). Systemically administered trefoil factors are secreted into the gastric lumen and increase the viscosity of gastric contents. Br. J. Pharmacol..

[28] Hanisch FG, Bonar D, Schloerer N, Schroten H (2014). Human trefoil factor 2 is a lectin that binds αGlcNAc-capped mucin glycans with antibiotic activity against *Helicobacter pylori*. J. Biol. Chem..

[29] Thim L, Madsen F, Poulsen SS (2002). Effect of trefoil factors on the viscoelastic properties of mucus gels. Eur. J. Clin. Invest..

[30] Hoffmann W (2015). TFF2, a MUC6-binding lectin stabilizing the gastric mucus barrier and more. Int. J. Oncol..

