# Supplementary material for: Decreased expression of TFF2 and decreased αGlcNAc glycosylation are malignant biomarkers of pyloric gland adenoma of the duodenum
Source: Sci Rep. 2023 Dec 8;13:21641. doi: 10.1038/s41598-023-49040-1 (PMC10703765; doi:10.1038/s41598-023-49040-1)
Supplement: Supplementary file 1 — Supplementary Information 1. [file 41598_2023_49040_MOESM1_ESM.pptx]

## Slide 1
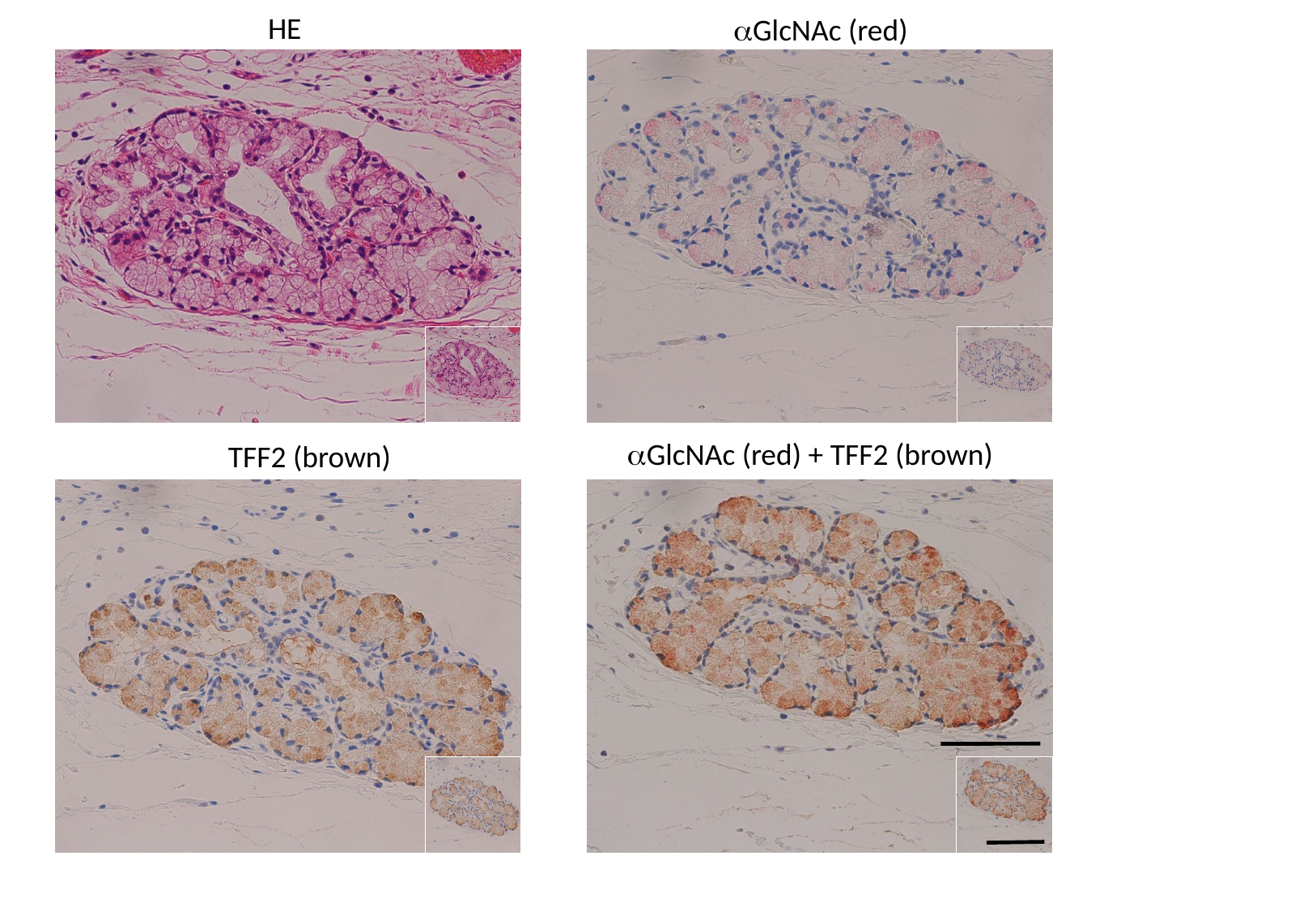

HE
aGlcNAc (red)
aGlcNAc (red) + TFF2 (brown)
TFF2 (brown)

## Slide 2
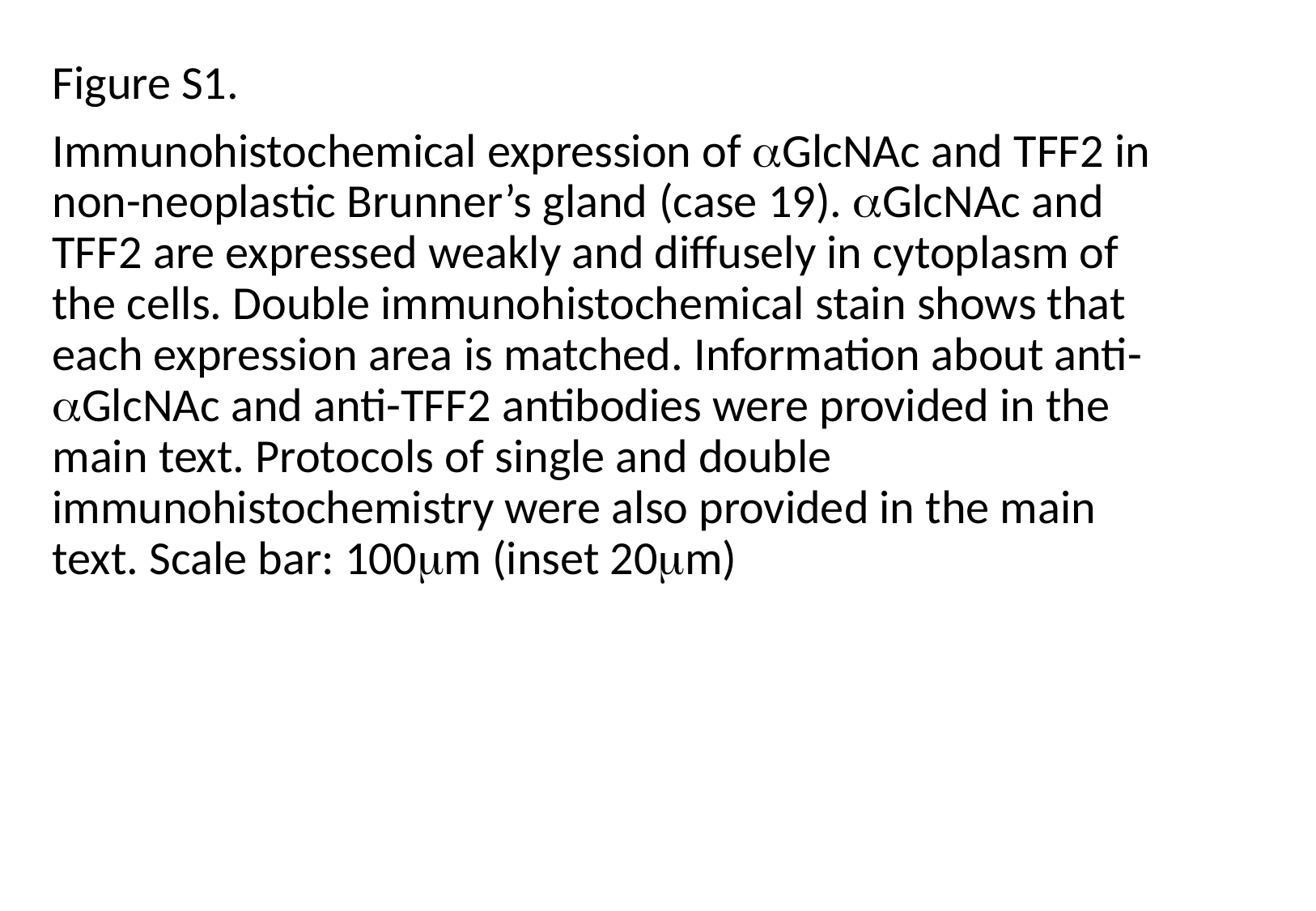

Figure S1.
Immunohistochemical expression of aGlcNAc and TFF2 in non-neoplastic Brunner’s gland (case 19). aGlcNAc and TFF2 are expressed weakly and diffusely in cytoplasm of the cells. Double immunohistochemical stain shows that each expression area is matched. Information about anti-aGlcNAc and anti-TFF2 antibodies were provided in the main text. Protocols of single and double immunohistochemistry were also provided in the main text. Scale bar: 100mm (inset 20mm)

## Slide 3
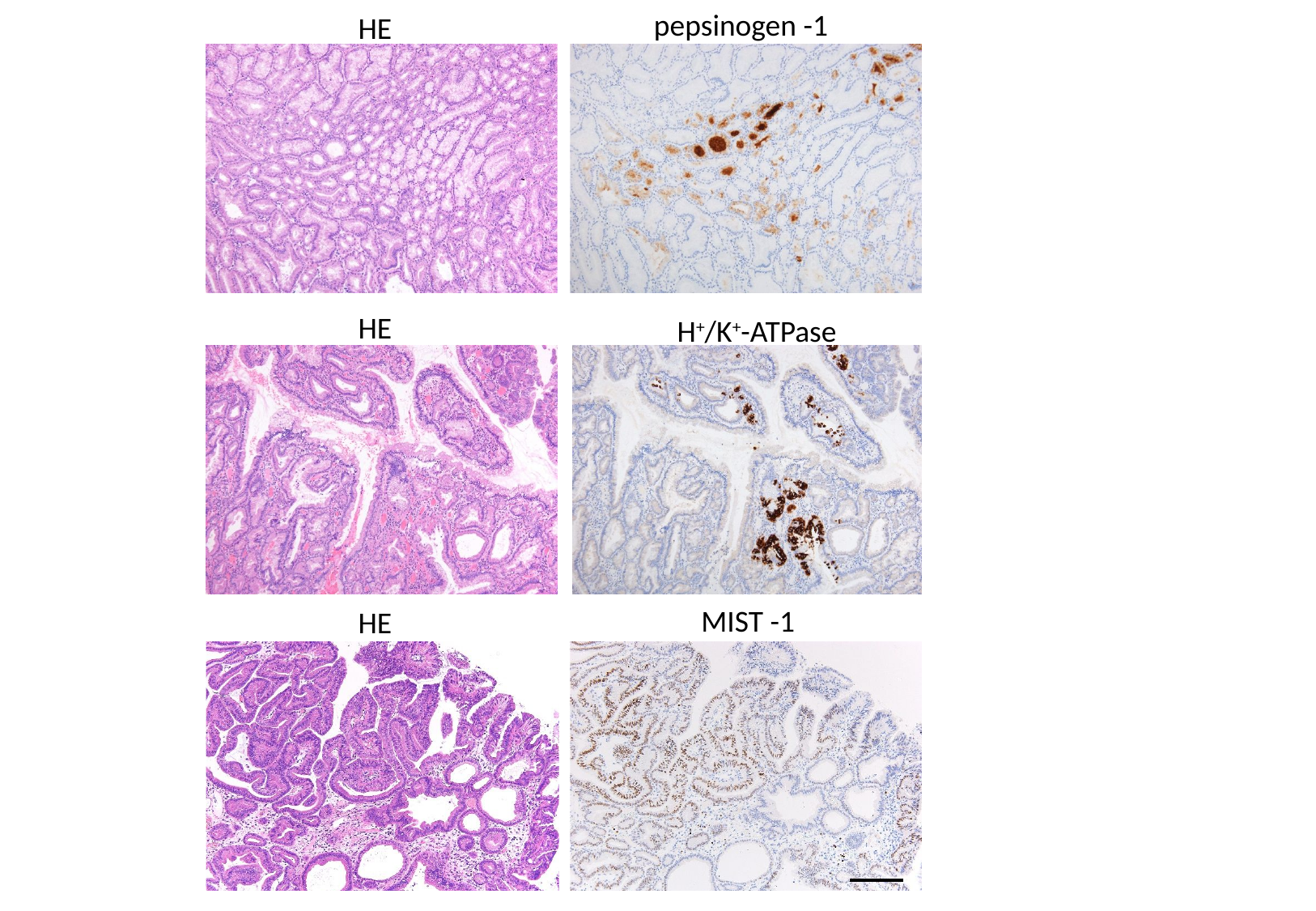

pepsinogen -1
HE
HE
H+/K+-ATPase
MIST -1
HE

## Slide 4
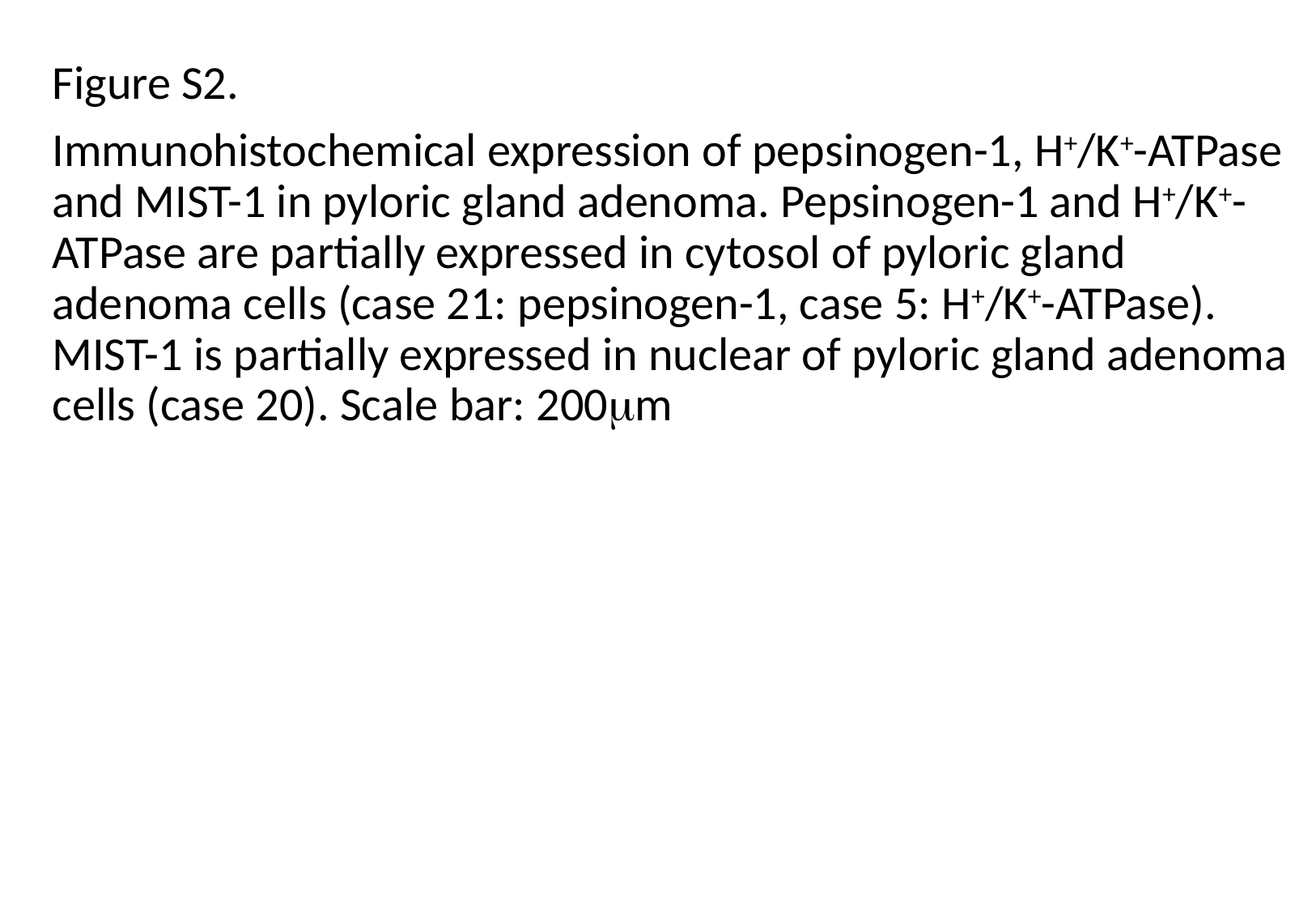

Figure S2.
Immunohistochemical expression of pepsinogen-1, H+/K+-ATPase and MIST-1 in pyloric gland adenoma. Pepsinogen-1 and H+/K+-ATPase are partially expressed in cytosol of pyloric gland adenoma cells (case 21: pepsinogen-1, case 5: H+/K+-ATPase). MIST-1 is partially expressed in nuclear of pyloric gland adenoma cells (case 20). Scale bar: 200mm
