# Supplementary material for: Decreased expression of TFF2 and decreased αGlcNAc glycosylation are malignant biomarkers of pyloric gland adenoma of the duodenum
Source: Sci Rep. 2023 Dec 8;13:21641. doi: 10.1038/s41598-023-49040-1 (PMC10703765; doi:10.1038/s41598-023-49040-1)
Supplement: Supplementary file 2 — Supplementary Information 2. [file 41598_2023_49040_MOESM2_ESM.doc]

**Supplement Table 1. Immunohistochemical characteristics of low grade pyloric gland adenoma component**s examined in this study

| Case No. | ^1^ Immunohistochemistry | | | | |  |
| --- | --- | --- | --- | --- | --- | --- |
|  | MUC2 | MUC5AC | MUC6 | αGlcNAc | TFF2 | p53 |
| 1 | 0 | 3 | 3 | 1 | 1 | Wild |
| 2 | 0 | 3 | 3 | 2 | 3 | Wild |
| 4 | 0 | 3 | 3 | 2 | 2 | Wild |
| 5 | 0 | 2 | 3 | 2 | 2 | Wild |
| 6 | 0 | 1 | 3 | 3 | 3 | Wild |
| 7 | 0 | 3 | 3 | 3 | 3 | Wild |
| 8 | 0 | 3 | 2 | 2 | 1 | Wild |
| 9 | 0 | 3 | 3 | 3 | 3 | Wild |
| 11 | 0 | 2 | 3 | 3 | 3 | Wild |
| 12 | 0 | 3 | 3 | 3 | 3 | Wild |
| 14 | 0 | 2 | 3 | 2 | 2 | Wild |
| 15 | 0 | 2 | 3 | 3 | 3 | Wild |
| 16 | 0 | 3 | 3 | 1 | 1 | Wild |
| 17 | 0 | 2 | 3 | 2 | 2 | Wild |
| 18 | 0 | 3 | 3 | 0 | 0 | Wild |
| 19 | 0 | 1 | 3 | 3 | 3 | Wild |
| 20 | 0 | 3 | 3 | 3 | 3 | Wild |
| 21 | 0 | 2 | 3 | 3 | 2 | Wild |
| 22 | 0 | 3 | 1 | 1 | 1 | Wild |
| 23 | 0 | 1 | 3 | 3 | 2 | Wild |

^1^0, < 10% of tumor cells; 1, 11 - 33% of tumor cells; 2, 33 - 66% of tumor cells; 3, > 66% of tumor cells.

**Supplement Table 2. Immunohistochemical characteristics of high grade pyloric gland adenoma component**s examined in this study

| Case No. | ^1^ Immunohistochemistry | | | | |  |
| --- | --- | --- | --- | --- | --- | --- |
|  | MUC2 | MUC5AC | MUC6 | αGlcNAc | TFF2 | p53 |
| 1 | 0 | 3 | 3 | 1 | 1 | Wild |
| 2 | 0 | 2 | 3 | 1 | 1 | Wild |
| 3 | 0 | 3 | 3 | 1 | 1 | Wild |
| 4 | 0 | 2 | 2 | 1 | 1 | Wild |
| 5 | 0 | 1 | 2 | 1 | 0 | Wild |
| 6 | 0 | 0 | 2 | 1 | 1 | Wild |
| 7 | 0 | 2 | 2 | 1 | 1 | Wild |
| 8 | 0 | 3 | 1 | 1 | 1 | Wild |
| 9 | 0 | 2 | 3 | 1 | 1 | Wild |
| 11 | 0 | 1 | 3 | 1 | 1 | Wild |
| 12 | 0 | 3 | 2 | 1 | 1 | Wild |
| 13 | 0 | 3 | 2 | 0 | 0 | Wild |
| 14 | 0 | 2 | 3 | 2 | 2 | Wild |
| 16 | 0 | 3 | 3 | 1 | 1 | Wild |
| 17 | 0 | 1 | 3 | 2 | 2 | Wild |
| 18 | 0 | 3 | 3 | 0 | 0 | Wild |
| 19 | 0 | 2 | 3 | 1 | 1 | Wild |
| 20 | 0 | 3 | 3 | 3 | 1 | Wild |
| 21 | 0 | 3 | 3 | 2 | 0 | Wild |
| 23 | 0 | 0 | 3 | 1 | 0 | Wild |

^1^0, < 10% of tumor cells; 1, 11 - 33% of tumor cells; 2, 33 - 66% of tumor cells; 3, > 66% of tumor cells.

**Supplement Table 3. Immunohistochemical expression score ratios (αGlcNAc/MUC6 and TFF2/MUC6)** of low grade pyloric gland adenoma components examined in this study

| Case No. | Immunohistochemical Expression Score Ratio | |
| --- | --- | --- |
| ^1^ αGlcNAc/MUC6 | ^2^ TFF2/MUC6 |
| 1 | 0.33 | 0.33 |
| 2 | 0.67 | 1.0 |
| 4 | 0.67 | 0.67 |
| 5 | 0.67 | 0.67 |
| 6 | 1.0 | 1.0 |
| 7 | 1.0 | 1.0 |
| 8 | 1.0 | 0.50 |
| 9 | 1.0 | 1.0 |
| 11 | 1.0 | 1.0 |
| 12 | 1.0 | 1.0 |
| 14 | 0.67 | 0.67 |
| 15 | 1.0 | 1.0 |
| 16 | 0.33 | 0.33 |
| 17 | 0.67 | 0.67 |
| 18 | 0.0 | 0.0 |
| 19 | 1.0 | 1.0 |
| 20 | 1.0 | 1.0 |
| 21 | 1.0 | 0.67 |
| 22 | 1.0 | 1.0 |
| 23 | 1.0 | 0.67 |

^1^ αGlcNAc/MUC6: Immunohistochemical expression score of αGlcNAc / Immunohistochemical expression score of MUC6, ^2^ TFF2/MUC6: Immunohistochemical expression score of TFF2 / Immunohistochemical expression score of MUC6,

**Supplement Table 4. Immunohistochemical expression score ratios (αGlcNAc/MUC6 and TFF2/MUC6) of high grade pyloric** gland adenoma components examined in this study

| Case No. | Immunohistochemical Expression Score Ratio | |
| --- | --- | --- |
| ^1^ αGlcNAc/MUC6 | ^2^ TFF2/MUC6 |
| 1 | 0.33 | 0.33 |
| 2 | 0.33 | 0.33 |
| 4 | 0.33 | 0.33 |
| 5 | 0.50 | 0.50 |
| 6 | 0.50 | 0.0 |
| 7 | 0.50 | 0.50 |
| 8 | 0.50 | 0.50 |
| 9 | 1.0 | 1.0 |
| 11 | 0.33 | 0.33 |
| 12 | 0.33 | 0.33 |
| 14 | 0.50 | 0.50 |
| 15 | 0.0 | 0.0 |
| 16 | 0.67 | 0.67 |
| 17 | 0.33 | 0.33 |
| 18 | 0.67 | 0.67 |
| 19 | 0.0 | 0.0 |
| 20 | 0.33 | 0.33 |
| 21 | 1.0 | 0.33 |
| 22 | 0.67 | 0 |
| 23 | 0.33 | 0 |

^1^ αGlcNAc/MUC6: Immunohistochemical expression score of αGlcNAc / Immunohistochemical expression score of MUC6, ^2^ TFF2/MUC6: Immunohistochemical expression score of TFF2 / Immunohistochemical expression score of MUC6,

**Supplement Table 5. Gastric fundic gland marker expression status of**

**pyloric gland adenoma** examined in this study

| Case No. | ^1^ Immunohistochemistry | | |  |
| --- | --- | --- | --- | --- |
|  | Pepsinogen-1 | H/K-ATPase | MIST-1 |  |
| 1 | 0 | 0 | 0 |  |
| 2 | 0 | 0 | 0 |  |
| 3 | 0 | 0 | 1 |  |
| 4 | 1 | 0 | 2 |  |
| 5 | 1 | 1 | 0 |  |
| 6 | 0 | 0 | 1 |  |
| 7 | 0 | 1 | 2 |  |
| 8 | 0 | 1 | 2 |  |
| 9 | 0 | 0 | 0 |  |
| 11 | 0 | 0 | 3 |  |
| 12 | 0 | 0 | 0 |  |
| 13 | 0 | 0 | 0 |  |
| 14 | 3 | 0 | 2 |  |
| 15 | 0 | 0 | 0 |  |
| 16 | 0 | 0 | 1 |  |
| 17 | 0 | 0 | 1 |  |
| 18 | 0 | 2 | 2 |  |
| 19 | 1 | 0 | 3 |  |
| 20 | 1 | 0 | 2 |  |
| 21 | 2 | 0 | 1 |  |
| 22 | 0 | 0 | 0 |  |
| 23 | 0 | 0 | 1 |  |

^1^0, < 10% of tumor cells; 1, 11 - 33% of tumor cells; 2, 33 - 66% of tumor cells;

3, > 66% of tumor cells.
